# Effect of saddle height on skin temperature measured in different days of cycling

**DOI:** 10.1186/s40064-016-1843-z

**Published:** 2016-02-27

**Authors:** Jose Ignacio Priego Quesada, Felipe P. Carpes, Rosario Salvador Palmer, Pedro Pérez-Soriano, Rosa Mª Cibrián Ortiz de Anda

**Affiliations:** Research Group in Sport Biomechanics (GIBD), Department of Physical Education and Sports, University of Valencia, Valencia, Spain; Biophysics and Medical Physics Group, Department of Physiology, University of Valencia, Avd/Blasco Ibañez 15, 46010 Valencia, Spain; Applied Neuromechanics Group, Laboratory of Neuromechanics, Federal University of Pampa, Uruguaiana, RS Brazil

**Keywords:** Infrared thermography, Thermal imaging, Exercise, Bike-fit, Reproducibility, Reliability

## Abstract

Infrared thermography can be useful to explore the effects of exercise on neuromuscular function. During cycling, it could be used to investigate the effects of saddle height on thermoregulation. The aim of this study was to examine whether different cycling postures, elicited by different knee flexion angles, could influence skin temperature. Furthermore, we also determined whether the reproducibility of thermal measurements in response to cycling differed in the body regions affected or not affected by saddle height. Sixteen cyclists participated in three tests of 45 min of cycling at their individual 50 % peak power output. Each test was performed in a different knee flexion position on the bicycle (20°, 30°, 40° knee flexion when the pedal crank was at 180°). Different knee angles were obtained by changing saddle height. Skin temperatures were determined by infrared thermography before, immediately after and 10 min after the cycling test, in 16 different regions of interest (ROI) in the trunk and lower limbs. Changes in saddle height did not result in changes in skin temperature in the ROI. However, lower knee flexion elicited higher temperature in popliteus after cycling than higher flexion (p = 0.008 and ES = 0.8), and higher knee flexion elicited lower temperature variation in the tibialis anterior than intermediate knee flexion (p = 0.004 and ES = 0.8). Absolute temperatures obtained good and very good intraday reproducibility in the different measurements (ICCs between 0.44 and 0.85), but temperature variations showed lower reproducibility (ICCs between 0.11 and 0.74). Different postures assumed by the cyclist due to different saddle height did not influence temperature measurements. Skin temperature can be measured on different days with good repeatability, but temperature variations can be more sensitive to the effects of an intervention.

## Background

Bike-fit represents the adjustment of the geometry of the bicycle and its components according to the dimensions of the cyclist body, with the general purpose of maximizing performance and reducing injury risk (Disley and Li 2014; Fonda et al. 2014). Saddle height is one of the most frequently assessed dimensions during bike-fit (Bini et al. 2011).

Different studies showed that changes in saddle height affect neuromuscular activation during cycling. Sanderson and Amoroso (2009) observed that greater knee flexion (differences of 17°) due to low saddle height decreased neuromuscular activation in the soleus and medial gastrocnemius. Jorge and Hull (1986) found lower activation of quadriceps and hamstring when saddle height was set at 95 % of trochanteric length compared to saddle height set at 100 %. These differences in the neuromuscular activation can theoretically affect the heat production of the muscles, thus affecting skin temperature (Saltin et al. 1970; Taylor 2000; Kenny et al. 2003). Indeed, these thermal effects could be even greater during aerobic cycling in hot environments, resulting in high skin temperatures that reduce the heat dissipation between the core and the skin (Sawka et al. 2012). This could increase core temperature to such high values (~40 °C) as to lead to fatigue and an increased risk of heat exhaustion and heat stroke (Nybo 2010; Sawka et al. 2012; Best et al. 2014).

The effects of the saddle height during cycling have been extensively examined through assessment of kinematics (Ferrer-Roca et al. 2012; Fonda et al. 2014), electromyography (Jorge and Hull 1986; Sanderson and Amoroso 2009), pedal forces (Iriberri et al. 2008; Bini et al. 2014), and gas exchange (Peveler and Green 2011). Considering the previously discussed relationship between muscle activation and heat production (Saltin et al. 1970; Kenny et al. 2003; Priego Quesada et al. 2015a), infrared thermography (IRT) can be an additional tool to explore the effects of saddle height during cycling from a thermal point of view. IRT is a technique that enables measurement of skin temperature, with valuable applications in the study of the thermal effects of physical exercise (Formenti et al. 2013; Abate et al. 2013). This technique provides some advantages to skin temperature measurement over other methods (e.g. thermocouples) since it is a non-invasive and non-contact technique with high sensitivity and accuracy (de Andrade Fernandes et al. 2014). However, to properly measure skin temperature it is necessary to control intervening factors (for details, see Fernández-Cuevas et al. 2015) in order to reduce variability. Variability of skin temperature data on different days can be greater than the effect of changes in saddle height on skin temperature. In addition, reproducibility can be lower in ROIs affected by different saddle heights. For this reason, when research is focused on exploring different conditions on different days, such as in the present study, it is important to consider the reproducibility of skin temperature. McCoy et al. (2011) observed excellent reproducibility between days for IRT images from the paraspinal region. However, Zaproudina et al. (2008) found moderate reproducibility in the trunk and poor reproducibility in the extremities. The authors suggested that this result was probably due to physiological variability of blood flow in the distal parts of the body (Zaproudina et al. 2008). These studies were performed under baseline conditions but reproducibility data after exercise is still necessary.

Here we set out to examine the influence of different cycling postures elicited by different knee flexion angles on skin temperature in response to cycling exercise. A secondary purpose was to determine the reproducibility of thermal measurements taken for specific regions of interest (ROI) after exercise performed on three different days. Different studies have shown differences in neuromuscular activation in quadriceps, hamstrings and gastrocnemius due to changes in saddle height (Jorge and Hull 1986; Sanderson and Amoroso 2009). For this reason, we hypothesized that higher knee flexion would reduce the skin temperature in the calf and in the hamstrings due a possible lower neuromuscular activation, and increase temperature in the quadriceps, due to possible higher neuromuscular activation, as reflected in the literature. Secondly, it was hypothesized that reproducibility on different days would be very good, so allowing a similar thermal baseline state, and that after-test reproducibility would not be affected by changes in saddle height (e.g. ROI of the trunk). However, poor reproducibility could be expected after exercise in the ROI affected by changes in the saddle height (calf, hamstrings and quadriceps).

## Methods

### Participants

Sixteen male cyclists categorized as club level, in accordance with the recommendations of Ansley and Cangley (2009), participated in this study. Mean (standard deviation): age [29 (10) years], body mass [77 (9) kg], height [178.7 (6.5) cm], average cycling training volume [230 (133) km/week], peak power output [273 (48) Watts]. Of the sixteen cyclists, one had a preferred left limb according to Waterloo Footedness Questionnaire (Elias et al. 1998). They all gave their written informed consent before participating. The study procedures complied with the Declaration of Helsinki, and were approved by the University’s ethics committee (approval number H1384344515519).

In order to measure the temperature of the participants’ skin under similar conditions, all of them were informed that they should not drink alcohol or smoke for at least 12 h before the test, should not carry out high-intensity or exhaustive exercise for at least 24 h before each test, and should avoid drinking coffee or other stimulants, avoid wearing any jewellery, sunbathing or being exposed to UV rays, as well as refraining from using sunscreen/sun blockers, body lotions and creams before the test. They should not eat for at least 2 h before the test. Finally, each participant was measured at the same time in the three tests performed in order to reduce the intra-subject effect of the circadian cycle.

### Protocol

The participants completed one pre-test and three main tests carried out on different days. The differences between the main tests were the knee flexion and extension amplitudes. All trials were performed on a stationary cycle ergometer (Cardgirus Medical, Bikemarc, Sabadell, Spain).

In the first visit, all participants performed an incremental cycling trial into determine maximal power output. This incremental trial consisted of 5-min warm-up at initial workload of 50 W, followed by sequential 1-min phases in which the workload was increased in steps of 25 W until exhaustion (Carpes et al. 2011). The pedaling cadence was controlled at 90 ± 3 rpm, and exhaustion was defined as the moment when the cyclist was no longer able to maintain a pedaling cadence of 87 rpm. Peak power output (PO_max_) was defined as the workload of the last phase completed. Posture and the saddle height during the incremental test were set at the same as on their own bicycles.

The three main tests began with a 3-min warm-up at 50 W and 90 rpm. Firstly, the participants stood in underpants for 10 min in order to adapt to the thermal room temperature before the IRT measurements. After this, participants put on their cycling clothes and shoes (same in the three tests). The participants then cycled for 45 min at 50 % POmax at 90 ± 2 rpm while maintaining a specific cycling posture. Each main test was performed with a specific knee flexion angle (40°, 30°, or 20°) when the pedal crank was at 180°, and the order of the tests was randomized. The knee angle was defined as the angle of knee flexion relative to the anatomical reference posture (static upright standing posture) taken as zero degrees (offset posture) (Peveler et al. 2012). Trunk flexion (maintained between 40° and 50° between the transverse plane and the union of the left acromion and the olecranon tuberosity), arm extension (maintained as a 75°–90° angle between the arms and the trunk), and the horizontal posture of the saddle, as defined by the plummet method (Zani 2010), were controlled throughout the tests. Environmental conditions during the test with the knee flexion angle at 40°, 30° and 20° were 23.4 ± 1.1 °C and 45.4 ± 12.5 %, 23.6 ± 1.2 °C and 40.7 ± 11.3 %, and 24.0 ± 1.2 °C and 50.8 ± 11.2 % relative humidity, respectively.

Posture was determined before each main test. Participants cycled at 50 W and 90 rpm, with knee flexion angle (obtained by changing the saddle height) using a 2D kinematic analysis system (IBV, Valencia, Spain) with a high-definition video camera sampling at 50 Hz (Sony Handycam HDR-FX1, Sony Corp., Tokyo, Japan). Reflective markers were attached to the lateral malleolus, lateral femoral condyle, greater trochanter, left acromion, and olecranon tuberosity from the left body side. A correction factor consisting of adding 2.2° to the measurements was performed (Fonda et al. 2014).

### Skin temperature measurement

Skin temperatures were determined in the main tests by IRT camera with infrared resolution of 320 × 240 pixels, thermal sensitivity <0.05 °C, and accuracy of ±2 °C (*FLIR E*-*60*, *Flir Systems Inc*., *Wilsonville*, *Oregon*, *USA*). A black body (BX-500 IR Infrared Calibrator, CEM, Shenzhen, China) was used to ensure a correct calibration of the camera. Thermal images of each participant were taken three times: before the cycling test and after 10 min of thermal adaptation to room temperature (Marins et al. 2014), immediately after the cycling test, and 10 min after finishing the cycling test. The images were recorded while the participant was standing up wearing underpants. With the aim of avoiding the effect of skin surface rubbing on skin measurement, sweat was not removed after the cycling test. The camera was positioned 1 m away from the participant and kept perpendicular to the body regions of interest.

Environmental conditions were controlled (e.g. lighting and temperature controlled room, no person apart from the investigator and the participant and no electronic equipment within a range of 5 meters). An anti-reflective panel was placed behind the participant to minimize effects from infrared radiation reflected in the wall (Hildebrandt et al. 2012). For all measurements, air temperature and relative humidity were measured using a thermo-hygrometer (Digital thermo-hygrometer, *TFA Dostmann, Wertheim*-*Reicholzheim, Germany*) and were computerized in the camera settings.

**Fig. 1:**
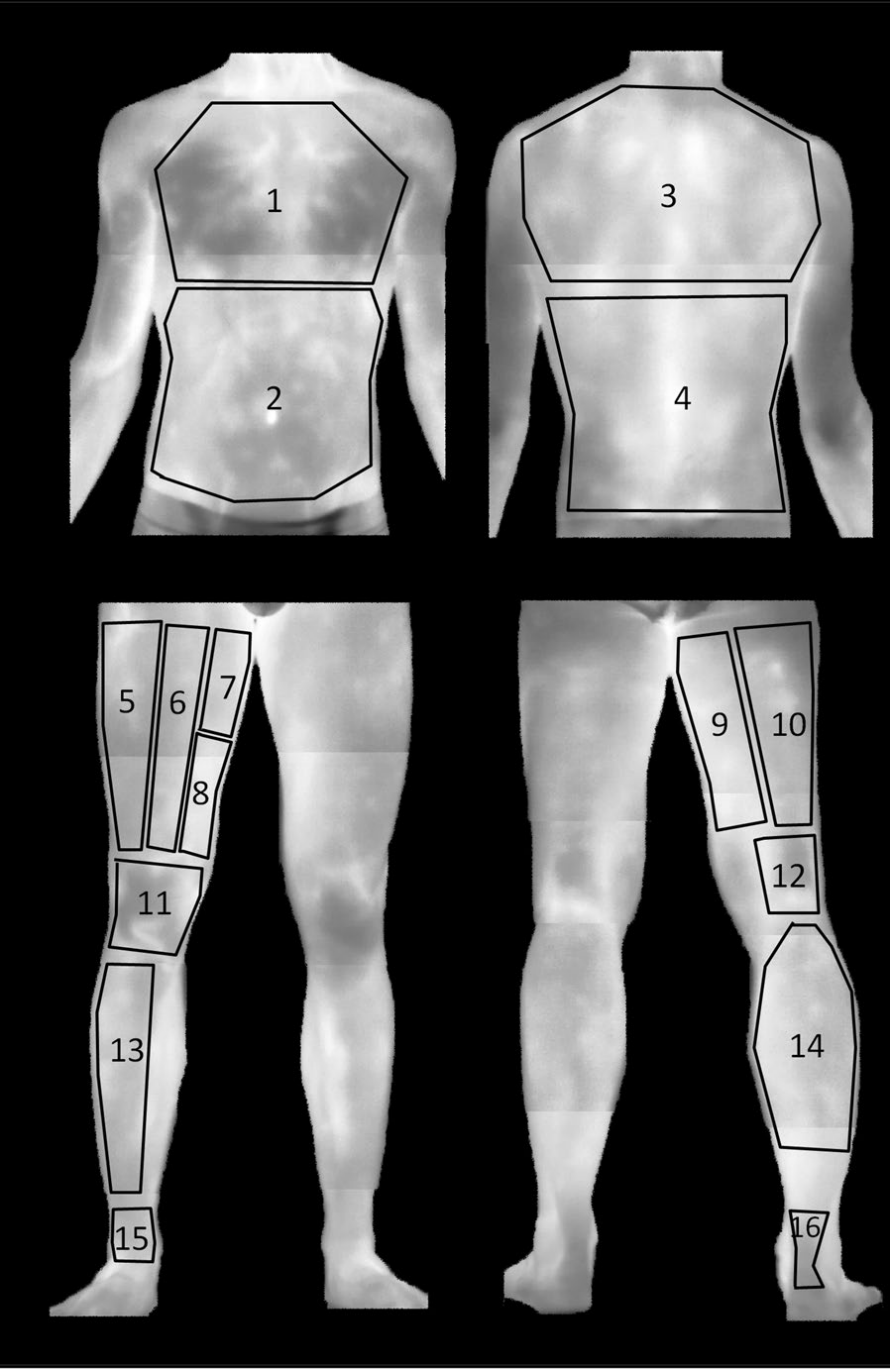
Representative illustration of the regions of interest (ROI): *1* chest, *2* abdomen, *3* upper back, *4* lower back, *5* vastus lateralis, *6* rectus femoris, *7* abductor, *8* vastus medialis, *9* biceps femoris, *10* semitendinosus, *11* knee, *12* popliteal, *13* tibialis anterior, *14* gastrocnemius, *15* ankle anterior, and *16* Achilles

Sixteen ROIs were defined (chest, abdomen, upper back, lower back, vastus lateralis, rectus femoris, abductor, vastus medialis, biceps femoris, semitendinosus, knee, popliteal, tibialis anterior, gastrocnemius, ankle anterior, and Achilles) (Fig. 1). Each ROI of a similar area was selected for all participants and for each time measurement. For the lower limbs, ROIs were selected in the preferred limb. Absolute mean temperature of each ROI was obtained using thermography software (Thermacam Researcher Pro 2.10 software, *FLIR, Wilsonville, Oregon, USA*), with an emissivity factor of 0.98 (Steketee 1973).

Skin temperature variation was assessed for each ROI based on the following variables (Priego Quesada et al. 2015a):

- ΔT: Difference between temperature before and immediately after the cycling test, expressed in °C.
- ΔT10: Difference between temperature before and 10 min after the cycling test, expressed in °C.
- ΔTafter: Difference between temperature immediately after and 10 min after the cycling test, expressed in °C.

### Statistical analysis

The statistics package SPSS 21 (SPSS Statistics, IBM) was used for the statistical analysis. The normality of each variable was confirmed by the Shapiro–Wilk test (p > 0.05). After this, differences between the three knee flexions (40°, 30°, and 20°) for absolute temperatures in each ROI were examined by applying two-way repeated measures ANOVA. For absolute temperatures, one-way repeated measures ANOVA were performed in each ROI. For both analyses, Bonferroni post hoc tests were used for pairwise comparison if applicable. Intra-class correlation coefficient (ICC) from model “2,1” (Shrout and Fleiss 1979) was calculated to determine the intraday reproducibility of each ROI. To assess reproducibility, the following classification of ICC values was used (Weir 2005): values 1.00–0.81 (excellent reproducibility), 0.80–0.61 (very good), 0.60–0.41 (good), 0.40–0.21 (reasonable) and, from 0.20–0.00 (poor). The typical error of the measurement was calculated to represent absolute consistency across the tests (Hopkins 2000; Weir 2005). Data are reported as mean ± SD with 95 % confidence intervals (95 % CI). Effect sizes (ES) (Cohen 1988) were calculated using a purpose-designed Excel spreadsheet (Microsoft Inc., USA). The level of statistical significance was taken to be p < 0.05 and ES > 0.8 (Cohen 1988).

## Results

### Effects of knee angle on skin temperature

Table 1 shows the temperature values obtained in each ROI corresponding to the different knee angle during cycling. Participants performed the three cycling tests at a specific knee flexion (40°, 30° and 20°) calculated from the offset posture. The absolute knee angle values for 40°, 30° and 20° were 50.4 ± 3.5°, 39.8 ± 4.0, and 30.0 ± 4.9, respectively.

**Table 1:** Absolute temperature values obtained from the different body ROIs (regions of interest) at the three measurement times in the three tests (20°, 30° and 40° knee flexion angle test)

ROI	Before cycling					Immediately after cycling					10 min after finishing cycling				
	Average ± SD			Reproducibility		Average ± SD			Reproducibility		Average ± SD			Reproducibility	
	20°	30°	40°	Typical error	ICC	20°	30°	40°	Typical error	ICC	20°	30°	40°	Typical error	ICC
Chest	32.8 ± 1.2	32.6 ± 0.9	32.6 ± 0.6	0.21	0.69	33.6 ± 0.8	33.2 ± 0.9	33.1 ± 1.2	0.22	0.63	32.6 ± 0.8	32.4 ± 1.1	32.5 ± 1.0	0.22	0.69
Abdomen	32.5 ± 1.5	32.3 ± 1.2	32.3 ± 0.9	0.28	0.8	32.2 ± 1.3	31.5 ± 1.4	31.5 ± 1.3	0.29	0.6	31.8 ± 1.2	31.5 ± 1.6	31.8 ± 1.3	0.31	0.75
Upper back	33.5 ± 1.0	33.4 ± 0.9	33.2 ± 0.7	0.19	0.71	33.6 ± 0.7	33.1 ± 1.1	33.1 ± 1.2	0.23	0.66	33.2 ± 0.9	33.2 ± 1.0	33.2 ± 0.9	0.22	0.73
Lower back	32.7 ± 1.4	32.5 ± 1.2	32.4 ± 0.8	0.25	0.7	33.4 ± 1.0	32.7 ± 2.0	32.5 ± 2.4	0.41	0.63	32.5 ± 1.3	32.3 ± 1.6	32.4 ± 1.5	0.34	0.79
Vastus lateralis	32.1 ± 1.0	31.3 ± 1.2	32.1 ± 1.0	0.21	0.44	32.5 ± 1.3	32.3 ± 1.6	32.2 ± 1.2	0.31	0.73	32.8 ± 1.0	32.9 ± 1.0	32.6 ± 0.6	0.19	0.6
Rectus femoris	31.7 ± 1.2	31.2 ± 1.3	31.3 ± 0.9	0.25	0.57	32.3 ± 1.3	32.2 ± 1.7	32.1 ± 1.2	0.32	0.69	32.7 ± 1.1	32.7 ± 1.0	32.4 ± 0.9	0.22	0.61
Abductor	31.8 ± 1.6	31.3 ± 1.4	31.6 ± 1.0	0.31	0.72	31.6 ± 1.6	31.7 ± 1.3	31.6 ± 1.0	0.31	0.66	32.1 ± 1.2	32.2 ± 1.1	32.0 ± 1.2	0.25	0.62
Vastus medialis	32.1 ± 1.3	31.6 ± 1.3	31.8 ± 0.8	0.26	0.65	32.9 ± 1.4	32.8 ± 1.5	32.7 ± 1.2	0.32	0.79	33.2 ± 1.0	33.2 ± 1.0	33.0 ± 0.8	0.21	0.74
Biceps femoris	32.1 ± 1.3	31.6 ± 1.3	31.8 ± 0.8	0.25	0.6	31.6 ± 1.0	31.2 ± 1.4	31.1 ± 1.0	0.25	0.64	32.3 ± 0.9	31.9 ± 1.1	31.9 ± 0.9	0.21	0.65
Semitendinosus	32.5 ± 1.3	31.9 ± 1.4	32.1 ± 0.8	0.27	0.66	32.0 ± 1.1	31.5 ± 1.5	31.4 ± 1.0	0.27	0.69	32.8 ± 0.9	32.4 ± 1.1	32.3 ± 0.8	0.22	0.68
Knee	30.9 ± 1.4	30.3 ± 1.8	30.4 ± 1.3	0.31	0.53	32.2 ± 2.0	31.6 ± 2.3	31.4 ± 2.3	0.53	0.85	32.1 ± 1.7	32.1 ± 1.8	31.8 ± 1.6	0.4	0.85
Popliteus	32.8 ± 1.1	32.3 ± 1.0	32.5 ± 0.7	0.2	0.63	*32.2* *±* 0.7*	31.9 ± 1.0	*31.6* *±* 0.7*	0.17	0.54	32.8 ± 0.8	32.7 ± 0.7	32.5 ± 0.6	0.15	0.61
Tibialis anterior	32.5 ± 1.2	32.0 ± 1.1	32.3 ± 0.8	0.2	0.62	31.5 ± 0.9	31.5 ± 1.1	31.1 ± 1.2	0.22	0.51	32.2 ± 0.7	32.4 ± 0.9	32.0 ± 0.8	0.17	0.62
Gastrocne-mius	32.5 ± 1.0	32.1 ± 0.9	32.2 ± 0.7	0.19	0.63	31.8 ± 0.9	31.6 ± 1.3	31.2 ± 1.0	0.25	0.7	32.6 ± 0.7	32.4 ± 0.9	32.1 ± 0.8	0.18	0.63
Ankle anterior	31.6 ± 1.2	30.9 ± 1.4	31.2 ± 0.8	0.24	0.5	32.1 ± 0.9	31.4 ± 2.0	31.3 ± 1.7	0.33	0.52	32.2 ± 0.8	31.9 ± 1.5	31.6 ± 1.2	0.26	0.65
Achilles	30.4 ± 1.4	29.6 ± 1.9	29.8 ± 1.0	0.31	0.5	31.1 ± 1.8	30.2 ± 2.3	29.9 ± 2.3	0.49	0.72	30.7 ± 1.5	30.0 ± 2.2	29.7 ± 1.9	0.43	0.71

Typical error and Intra-class correlation coefficient (ICC) was calculated to determine the intraday reproducibility

Statistical significant difference was observed in popliteus between the knee at 20° and 40° (p < 0.05) and it is indicated with italics letters and *

In the analysis of the absolute temperatures in the ROI, differences were obtained only in the popliteus (Table 1). Immediately after the cycling test, knee flexion at 20° elicited higher temperature in popliteus than knee flexion at 40° (20° vs 40°: 32.2 ± 0.7 vs 31.6 ± 0.7 °C, p = 0.008 and ES = 0.8, 95 % CI of the difference between conditions [0.1, 0.9 °C]). No differences were obtained for absolute temperatures between the three flexion angles in the other fifteen ROIs (p > 0.05 and ES < 0.8).

In the temperature variation analysis, differences were observed only in the tibialis anterior (Table 2). Knee flexion at 30° elicited higher ΔT10 in the tibialis anterior than knee flexion at 20° (30° vs 20°: 0.3 ± 0.9 vs −0.2 ± 0.8 °C, p = 0.004 and ES = 0.8, 95 % CI of the difference between conditions [0.2, 1.1 °C]). No differences were found between the three flexion angles for temperature variation in the other fifteen ROIs (p > 0.05 and ES < 0.8).

**Table 2:** Temperature variations values obtained from the different body ROIs (regions of interest) at the three measurement times in the three tests (20°, 30° and 40° knee flexion angle test)

ROI	ΔT					ΔT10					ΔTafter				
	Average ± SD			Reproducibility		Average ± SD			Reproducibility		Average ± SD			Reproducibility	
	20°	30°	40°	Typical error	ICC	20°	30°	40°	Typical error	ICC	20°	30°	40°	Typical error	ICC
Chest	0.8 ± 1.3	0.6 ± 1.2	0.5 ± 1.5	0.3	0.74	−0.2 ± 1.1	−0.3 ± 1.2	−0.1 ± 1.1	0.24	0.67	−1.0 ± 0.8	−0.8 ± 1.1	−0.6 ± 1.1	0.22	0.67
Abdomen	−0.3 ± 1.3	−0.7 ± 1.4	−0.8 ± 1.3	0.28	0.56	−0.6 ± 0.9	−0.8 ± 1.2	−0.5 ± 1.0	0.21	0.46	−0.4 ± 1.1	−0.1 ± 0.9	0.3 ± 1.0	0.22	0.63
Upper back	0.1 ± 1.3	−0.3 ± 1.4	−0.2 ± 1.5	0.32	0.73	−0.3 ± 1.2	−0.2 ± 1.1	−0.1 ± 1.0	0.24	0.61	−0.4 ± 0.7	0.1 ± 0.8	0.1 ± 0.8	0.17	0.6
Lower back	0.7 ± 1.5	0.3 ± 2.2	0.1 ± 2.6	0.48	0.7	−0.2 ± 1.6	−0.2 ± 1.6	−0.0 ± 1.5	0.35	0.69	−0.9 ± 0.8	−0.4 ± 0.9	−0.1 ± 1.7	0.24	0.43
Vastus lateralis	0.7 ± 1.2	1.0 ± 0.9	0.8 ± 0.9	0.22	0.62	1.0 ± 1.0	1.6 ± 0.8	0.6 ± 0.8	0.14	0.17	0.3 ± 0.7	1.2 ± 0.6	0.5 ± 0.7	0.16	0.52
Rectus femoris	0.6 ± 1.2	1.0 ± 0.9	0.7 ± 0.9	0.21	0.14	1.0 ± 1.0	1.5 ± 0.8	1.1 ± 0.7	0.14	0.11	0.4 ± 0.6	0.5 ± 0.8	0.4 ± 0.6	0.14	0.62
Abductor	−0.2 ± 1.1	0.4 ± 1.0	0.0 ± 0.8	0.18	0.33	0.3 ± 1.0	0.9 ± 0.9	0.5 ± 0.7	0.15	0.14	0.5 ± 0.7	0.5 ± 0.7	0.5 ± 0.6	0.13	0.54
Vastus medialis	0.8 ± 1.2	1.1 ± 0.8	0.9 ± 0.8	0.2	0.6	1.1 ± 1.1	1.5 ± 0.7	1.3 ± 0.5	0.14	0.22	0.3 ± 0.7	0.4 ± 0.8	0.3 ± 0.7	0.16	0.68
Biceps femoris	−0.5 ± 1.3	−0.4 ± 0.9	−0.8 ± 0.9	0.19	0.26	0.2 ± 1.0	0.4 ± 0.7	0.1 ± 0.7	0.15	0.32	0.7 ± 0.7	0.8 ± 0.7	0.9 ± 0.6	0.14	0.56
Semitendinosus	−0.5 ± 1.2	−0.5 ± 1.0	−0.7 ± 0.9	0.21	0.47	0.3 ± 0.8	0.4 ± 0.8	0.2 ± 0.7	0.15	0.44	0.8 ± 0.7	0.9 ± 0.7	0.9 ± 0.5	0.14	0.66
Knee	1.3 ± 2.0	1.4 ± 2.1	1.0 ± 2.3	0.48	0.73	1.3 ± 1.7	1.8 ± 1.8	1.4 ± 1.4	0.34	0.52	−0.1 ± 0.8	0.4 ± 0.8	0.4 ± 1.0	0.19	0.6
Popliteus	−0.6 ± 0.9	−0.4 ± 0.7	−0.8 ± 0.6	0.14	0.32	−0.0 ± 0.6	0.3 ± 0.7	0.0 ± 0.4	0.09	0.11	0.6 ± 0.5	0.8 ± 0.6	0.8 ± 0.4	0.09	0.27
Tibialis anterior	−1.0 ± 1.3	−0.6 ± 1.1	−1.2 ± 1.0	0.24	0.53	*−0.2* *±* 0.8*	*0.3* *±* 0.9*	−0.3 ± 0.6	0.16	0.45	0.7 ± 0.8	0.9 ± 0.6	0.9 ± 0.7	0.14	0.54
Gastrocnemius	−0.8 ± 0.9	−0.6 ± 0.8	−1.0 ± 1.0	0.18	0.45	0.1 ± 0.6	0.3 ± 0.6	−0.1 ± 0.6	0.11	0.21	0.9 ± 0.6	0.9 ± 0.5	1.0 ± 0.5	0.1	0.36
Ankle anterior	0.5 ± 1.4	0.5 ± 1.6	0.0 ± 1.6	0.33	0.58	0.6 ± 1.1	1.0 ± 1.3	0.3 ± 1.0	0.23	0.51	0.1 ± 0.8	0.5 ± 0.9	0.3 ± 0.7	0.16	0.38
Achilles	0.6 ± 1.6	0.7 ± 2.1	0.1 ± 1.8	0.39	0.57	0.3 ± 1.4	0.5 ± 1.9	−0.1 ± 1.4	0.33	0.53	−0.3 ± 0.6	−0.2 ± 0.8	−0.2 ± 0.7	0.14	0.45

Typical error and Intra-class correlation coefficient (ICC) was calculated to determine the intraday reproducibility

Statistical significant difference was observed in tibialis anterior between the knee at 20° and 30° (p < 0.05) and it is indicated with italics letters and *

### Intraday reproducibility

Table 1 shows the intra-class correlation coefficients of the absolute temperatures obtained from the three tests. Before the cycling test, the different ROIs presented good and very good reproducibility. Immediately after cycling, the different ROIs continued showing good and very good reproducibility. Moreover, the ROI of the knee showed excellent reproducibility. Ten minutes after the cycling test all ROIs presented ICC values higher than 0.6 (very good reliability).

Table 2 shows the temperature variations and their intra-class correlation coefficients. The ROI of the trunk presented good and very good reproducibility in the three temperature variations. However, the ROIs of the lower limbs presented lower values of ICC in temperature variations than in absolute values. ΔT values presented, in some ROIs, poor (rectus femoris) and reasonable reproducibility (abductor, biceps femoris, and popliteus). This tendency increased for ΔT10 values, more ROIs showing poor (vastus lateralis, rectus femoris, abductor and popliteus) and reasonable reproducibility (vastus medialis, biceps femoris and gastrocnemius). In the ΔTafter values, almost all ROIs of the limbs presented good reproducibility, and only three presented reasonable ICC values (popliteus, gastrocnemius and ankle anterior).

## Discussion

The purpose of the present study was to analyse the effect of knee flexion (elicited by different saddle heights) on skin temperature after cycling, and also to determine the reproducibility of skin temperature taken at specific ROI in the three different tests performed by cyclists. The main findings were that the ROI with possible changes in neuromuscular activation produced by the saddle height did not affect skin temperature. However, greatest knee extension presented higher temperature in the popliteus after cycling than the greatest knee flexion, and the greatest knee flexion elicited lower temperature variation (ΔT10) in the tibialis anterior than intermediate knee flexion. Furthermore, absolute temperatures obtained good and very good reproducibility at the different measurement times, but temperature variations of the lower limbs presented lower reproducibility.

We hypothesized the effects of different knee angles and saddle height positions on skin temperature, but no differences were observed. Although changes in the saddle height can increase the neuromuscular activation of specific muscles and thus increase their heat production (Saltin et al. 1970; Kenny et al. 2003), these were not reflected in increased skin temperature, probably due to higher sweat rate (Buono et al. 2010; Fujii et al. 2014). Higher sweat rate reduces the skin temperature and this favours its thermal gradient with the core (Cuddy et al. 2014). Thus, the result of the skin temperature is the balance between metabolic heat production and heat dissipation (González-Alonso 2012).

Thermal effects produced by the different knee flexions were observed in poplitius and tibialis anterior. The greatest knee extension (20° when the pedal crank is at 180°) showed a higher temperature in popliteus (ranged from 0.1 to 0.9 °C) than in the greatest knee flexion (40°). Different authors have associated pain in popliteus with a too high saddle height (Callaghan 2005; Silberman 2013). It is possible that the higher tendon elongation produced by the greatest knee extension results in a higher tendon blood volume (Kubo et al. 2009), and then an increase in skin temperature (Hildebrandt et al. 2012). On the other hand, the greatest knee flexion (40°) presented lower temperature variation (ΔT10, ranged between 0.2 and 1.1 °C) in the tibialis anterior than intermediate knee flexion (30°). Tibialis anterior is an ankle stabilizer during pedaling (So et al. 2005). A recent study observed lower range of ankle motion in an optimal saddle height (25°) than in low saddle height (45°) (Bini et al. 2014). These results were in agreement with the present study, in which the lowest increase in the tibialis anterior skin temperature was found between the greatest (40°) and the intermediate knee flexion (30°). Lower range of ankle motion can result in lower muscular activation of tibialis anterior (So et al. 2005), resulting in a lower temperature variation. However, although higher differences between the greatest and the lowest knee flexion (20°) could be expected, no differences were found between both postures. Further studies are necessary to explain and to validate the thermal differences between knee flexion in the popliteus and tibialis anterior.

The intraday reproducibility results obtained before cycling test in absolute temperature were better, except in the ROI of the knee, than those presented by Zaproudina et al. (2008). They presented a 0.76 ICC value for the trunk anterior (0.69 and 0.80 in chest and abdomen in the present study), 0.32 in the back (0.71 and 0.70 in upper and lower back) 0.42 in the thigh (0.60) 0.76 in the knee (0.53), and 0.52 in the calf (0.63). Differences between the results may be due to a number of reasons: differences in the ROI identification, different room adaptation (10 min lower in the present study) or/and differences in the variability of the blood flow of the participants (Zaproudina et al. 2008). In any case, IRT measurement has presented good and very good reproducibility following the rigorous methodology used by different authors and organizations (Ring and Ammer 2000; Ammer 2008; Hildebrandt et al. 2012; Priego Quesada et al. 2015a). Furthermore, the present study showed that reproducibility is still good after exercise. However, the temperature variation analysis showed similar reproducibility in the trunk, but worse in the lower limbs. This may be due to the fact that temperature variations can be more sensitive to changes in the saddle height than absolute temperatures. Hence, different studies have shown significant temperature variations after their interventions, but no differences in absolute temperatures (Formenti et al. 2013; Priego Quesada et al. 2015b). Similarly, different studies focus their significant results on temperature variations, probably because absolute temperatures do not reflect the effect of their interventions (Bertmaring et al. 2008; Chudecka and Lubkowska 2010). Further studies should investigate the reproducibility of temperature data after exercise without any intervention and whether temperature variations of the lower limbs present higher intraday reproducibility without changes in the saddle height or another type of intervention.

One limitation of the present study was that neuromuscular activation was not measured during the cycling test. Surface electromyography could show differences in the neuromuscular activation due to different saddle heights as reported by the literature (Jorge and Hull 1986; Sanderson and Amoroso 2009).

Sweat on the skin surface after the cycling tests could have influenced the IRT data and it should also be considered a limitation of the present study. A film of water on the skin may work as a filter for infrared radiation and, therefore, it could lead to an underestimation of the thermal data of the infrared measures (Ammer 2009). However, the studies undertaken are insufficient to clarify the effect of sweat on skin emissivity in infrared measures. For this reason, it was decided not to remove the sweat on the skin for the thermographic measurements after the cycling tests in order to avoid an increase of skin temperature due to rubbing, and also the reduction of the natural process of sweat evaporation (Priego Quesada et al. 2015b).

The present study assessed the effects on skin temperature after cycling 45 min at 50 % of the POmax. This intensity was chosen with the aim of carrying out a moderate aerobic intensity test. Future studies could explore if cycling tests with higher workload, but of shorter duration, could increase neuromuscular differences between knee flexions and later skin temperature.

## Conclusions

Different knee flexions, after 45 min of cycling, did not present differences in skin temperature in the ROI linked to changes in the neuromuscular activation reported in the literature. This finding demonstrates that the application of skin temperature analysis using IRT for studying the effects of different saddle heights does not appear to be suitable. However, reproducibility of the absolute temperatures after exercise on different days was good, temperature variations being more sensitive to the effects of an intervention. This means that skin temperature can be measured on different days by IRT, but temperature variation analysis may be better for studying the effect of an intervention. Future studies are needed to validate this last hypothesis.
